# Microglia dynamic response and phenotype heterogeneity in neural regeneration following hypoxic-ischemic brain injury

**DOI:** 10.3389/fimmu.2023.1320271

**Published:** 2023-11-29

**Authors:** Hongxin Quan, Runrui Zhang

**Affiliations:** ^1^State Key Laboratory of Primate Biomedical Research, Institute of Primate Translational Medicine, Kunming University of Science and Technology, Kunming, Yunnan, China; ^2^Yunnan Key Laboratory of Primate Biomedical Research, Kunming, Yunnan, China

**Keywords:** hypoxic-ischemic brain injury, microglia heterogeneity, microglia dynamic response, neural regeneration, neurogenesis

## Abstract

Hypoxic-ischemic brain injury poses a significant threat to the neural niche within the central nervous system. In response to this pathological process, microglia, as innate immune cells in the central nervous system, undergo rapid morphological, molecular and functional changes. Here, we comprehensively review these dynamic changes in microglial response to hypoxic-ischemic brain injury under pathological conditions, including stroke, chronic intermittent hypoxia and neonatal hypoxic-ischemic brain injury. We focus on the regulation of signaling pathways under hypoxic-ischemic brain injury and further describe the process of microenvironment remodeling and neural tissue regeneration mediated by microglia after hypoxic-ischemic injury.

## 1 Introduction

The brain, as the command center of the body, relies on a constant supply of blood and oxygen to maintain its proper function. Cerebral blood flow accounts for roughly 15-20% of cardiac output, while brain oxygen consumption represents around 20% of the total of the body (1). Therefore, the brain is more sensitive to changes in blood and oxygen than other organs. Hypoxic-ischemic injury in the brain may seriously affect central nervous system (CNS) niche homeostasis. Numerous studies have demonstrated that hypoxic-ischemic brain injury leads to pathological processes such as energy failure, oxidative stress, blood-brain barrier disruption, microglia activation inflammation, neuro-excitotoxicity, and endothelial damage (2–5). As the immune cells that reside in the brain, microglia patrol the brain parenchyma to maintain CNS niche homeostasis via phagocytosis and interaction with many other cells, such as neurons, astrocytes, oligodendrocytes, and endothelial cells (6–8). When brain tissue homeostasis is disrupted, microglia undergo a dynamic process to adjust their morphologies, phenotypes, and functions to respond promptly to different stimulations. This process is a response mechanism for microglia to adapt to environmental changes (9). Interestingly, numerous research studies have provided evidence that there is neurogenesis occurring in the altered environment (10–13). However, the potential mechanisms driving this process of neurogenesis remain unclear, which has sparked interest in understanding the role played by microglia in this process. An increasing number of viewpoints suggest that microglia play a beneficial role in neural regeneration (14–16). Therefore, understanding the dynamic and molecular changes that occur in microglia during injury is crucial for unraveling the contribution of microglia to neural regeneration. This review summarizes the dynamic changes of microglia in hypoxic-ischemic brain injury and primarily focuses on the specific responses, transformations, and pathway activations of microglia in this condition. The process of microenvironment remodeling and neural tissue regeneration after hypoxic-ischemic brain injury mediated by microglia cells was further described.

## 2 Microglia dynamic response and phenotype heterogeneity in hypoxic-ischemic related diseases

The microglia show great differences in morphology. Traditionally there are “ramified microglia” (have highly ramified processes and plays a certain function under normal physiological conditions), “reactive microglia” (have a rounded cell body with a few ramified processes) and “ameboid microglia” (display a characteristic amoeboid-shaped cell body extending one or two unramified processes or are completely devoid of processes) (17). Specifically, in the microenvironment of the steady-state, microglia have more ramified processes, and this “ramified microglia” monitors the dynamic balance of the microenvironment in real time (18). When the balance of the microenvironment is disturbed, microglia transform into “reactive microglia”, which are morphologically more rounded cell bodies with fewer and shorter ramified processes that respond to changes in the microenvironment and make immune responses (19, 20). With increased cell division and self-proliferation, reactive microglia change their morphological and molecular characteristics, and these microglia adopt amoeba shape and exhibit enhanced mobility, which allows them to efficiently migrate to areas of injury or disease within the CNS (21, 22), which is critical for their immune function and ability to clear cell debris (23). However, it is not comprehensive to judge the state and related functions of microglia only from morphology. For example, in adult hippocampal neurogenesis, ramified microglia can also perform phagocytosis (24).

Microglia exhibit various phenotypes in response to environmental stimuli, which are identified based on the types of factors they secrete and their specific physiological characteristics, resulting in substantial heterogeneity among these cells. The earlier research commonly categorized polarized microglia into M1 and M2 subtypes (25–27). M1 phenotypes are known as “classical” activation, which is typically induced by interferon-γ (IFN-γ) and lipopolysaccharide (LPS). M1 phenotypes have pro-inflammatory activity. They secrete various immunoreactive substances such as tumor necrosis factor-α (TNF-α), interleukin 6 (IL-6), and NO to neutralize viruses or bacteria, and stimulate the inflammatory response of other cells, thus causing specific immune resistance (26). The M2 “alternative” phenotype is an active anti-inflammatory state, which appears to regulate immunity. Its primary function is maintaining a stable niche balance and preventing excessive inflammation caused by the specific immune response (28). Based on the subtype classification of M2, Ma et al. further divided it into M2a, M2b, and M2c subtypes (29). M2a microglia are induced by IL-4 or IL-13 stimulation, also known as “alternative activated microglia”, and they play a key role in suppressing inflammation and facilitating tissue repair processes. Notably, in M2a microglia, the expression levels of Fizz1, Arg1, and Ym1 mRNA are upregulated (30). After traumatic spinal cord injury, M2a microglia support the intrinsic repair and recruitment of peripheral myeloid cells and promote recovery after injury (31). M2b phenotype is “transitionally activated microglia” that is induced by most pro-inflammatory stimuli LPS, IL-1β, TNF-α, or IFNγ and M2b phenotype is associated with immune regulation (32). M2c subtype is microglia exposed to IL-10 or transforming growth factor-β (TGF-β) or glucocorticoid induces a specific phenotype in microglia, also known as “acquired deactivated microglia”. The signature of this phenotype is involved in neuroprotection and releases a number of anti-inflammatory cytokines with high expression of IL-4Rα, CCR2, SOCS3, CD150 and CD206 (33). Meanwhile, the study has found that the response phenotypes of microglia are dynamic, and some of response phenotypes can be reversed (34). While some studies have discovered that microglia can improve symptoms when induced into an M2 phenotype, M2 does not wholly represent the beneficial neuroprotective subtype. The polarity of M1 and M2 microglia was defined in the pre-genomic era based on the expression of some markers and the function of the cell. However, the definition fails to detail the dynamic transcription of microglia during their response to stimuli (35). With the development of genome-wide transcriptomics and epigenomics as well as two-photon imaging techniques, microglia cells have been classified into more subtypes with different expression lineages (36, 37). For example, the Trem-2-dependent disease-associated microglia (DAMS) subpopulation discovered by karen-shaul et al. in a mouse model of Alzheimer’s disease (AD) via single-cell RNA-seq and smFISH (38). Interestingly, microglia of this subtype can be polarized in two steps, allowing them to acquire a protective phenotype in the context of neurodegeneration (39, 40). Similarly, the single-cell RNA-seq technique has been used to reveal the diverse transcriptional landscape of microglia in stroke, which is divided into more subtypes (41). As emphasized by leading scientists in the field, the states of microglia are intricately linked to the surrounding microenvironment. Microglial behavior is far from static due to their sophisticated regulatory mechanisms. Attempts to classify microglial subtypes unilaterally proved to be imperfect. To comprehensively define microglial states, one must consider their specific contexts across various dimensions (42). It is important to acknowledge that the category of microglia into M1 and M2 subtypes is not perfect. Microglia can exhibit a range of activation states beyond the M1 and M2 categories, and these states can dynamically transform and coordinate to play distinct roles in response to various stimuli. Overemphasizing the M1 and M2 categories may oversimplify the complexity of microglial activation and overlook the molecular and functional dynamics involved. Especially in diseases related to hypoxic-ischemic injury, the response of microglia is a dynamic process that is influenced by factors such as the duration and severity of hypoxia ischemia within the CNS (43–45). Understanding the diverse transcriptional landscape and the dynamic changes of microglia subtypes in hypoxic-ischemic injury is crucial for developing effective therapeutic strategies. In the following, we describe microglia dynamic response and phenotype heterogeneity under three typical hypoxic-ischemic brain diseases, including stroke, chronic intermittent hypoxia (CIH), and neonatal hypoxic-ischemic brain injury (NHIBI).

### 2.1 Stroke

Stroke is the second most common cause of death and the leading cause of disability worldwide. A stroke can cut off the blood supply to brain areas, leading to death or permanent neurological impairment (46). When the blood supply is disrupted, brain cells such as neurons, glial cells, and endothelial cells are also deprived of oxygen and energy (47–49). These cells may have undergone complex metabolic pathway changes, leading to the interaction of multiple signal transduction pathways and the inhibition of oxidative phosphorylation and ATP (Adenosine triphosphate) synthesis processes (46). During the complex pathophysiological process, irreversible damage was inflicted upon the brain. Microglia respond rapidly as niche homeostasis monitors (50). Microglia respond differentially during different stages of stroke.

During the acute phase of ischemic anoxic stroke, neurons in the infarct core region suffer from oxygen-glucose deprivation. Microglia respond rapidly to acute stroke stimulation (51). Interestingly, Guo et al. investigated microglial diversity and functional variations during the early stage of stroke by analyzing microglia collected 24 hours after stroke induction using the Middle cerebral artery occlusion (MCAO) model. They identified 14 subclusters of microglia and found that certain subclusters exhibited distinct functional differences in their early response to stroke. In these subclusters, genes related to phagocytosis (Id2, Cd83, Gadd45b, Ccl4, Rcan1) and inflammatory response (TNF-α, IL-6, IL-2) were upregulated (45). Additionally, the expression of inflammatory response related genes, such as iNOS, Cd11b, Cd16, Cd32 and Cd86, were all up-regulated (52). It has also been found that a distinct group of microglial cells associated with stroke injuries are significantly more abundant during this period and may play a protective role in ischemic wounds. These microglia exhibit significantly increased expression levels of SPP1, Itga5, Cd63 and Ftn1 (53). These findings suggest that within the first 24 hours after stroke, microglial function mainly manifested in inflammatory response.

During the sub-acute phase of stroke, there is a transition in the phenotypic characteristics of microglia from a pro-inflammatory state to an anti-inflammatory state. Specifically, microglia undergo significant proliferation during the subacute phase (54). Deng et al. showed that the microglia are more prone to anti-inflammatory predominance on day 3 and day 14 after hypoxic-ischemic stroke. They compared the 3-month and 12-month samples and found that the overall microglia activation was more significant in the younger samples, including microglia activation related Gpnmb, Lgals3 and proliferation-related Mki13 gene up-regulation (55). Shi et al. found that genes related to inflammatory response (Cspg4, Cst7, Chst2, Cxcl10), chemokines (Ccl2, Ccl3, Ccl4, Ccl6, Ccl8, Ccl12, Ccl10, Ccl16), and neural repair (SPP1, Cspg4, Gpnmb) were up-regulated in microglia subtypes during this period. Additionally, microglia from aged mice showed significantly reduced migration and intercellular interaction when compared to polarized microglia from young mice (56). In summary, during the sub-acute phase of stroke, microglia exhibit a shift towards anti-inflammatory predominance, accompanied by increased proliferation and specific gene expression patterns related to inflammation, chemokines, and neural repair.

During the chronic phase of stroke, the subtypes of microglia are mainly involved in neurovascular repair and brain tissue remodeling, and play a role in neural protection. For example, the significant changes in microglial gene expression levels occur such as up-regulation of SPP1, Cst7, Lgals3bp, Lpl and Igfbp5. Microglia in aged samples treated with MCAO exhibited a significant down-regulation of functions associated with cell motility, inflammatory response, cell viability, and cell homeostasis, as compared to their younger counterparts treated with MCAO (57). In summary, these studies provide additional insight into the transcriptional changes of polarized microglia in stroke and their corresponding functions. It is important to note that the secretion of inflammatory and regulatory factors by polarized microglia plays a crucial role in the regulation of the CNS niche. Nonetheless, the precise regulatory mechanisms of these factors are still unknown, emphasizing the need for further research.

### 2.2 Chronic intermittent hypoxia

CIH is a condition of intermittent apnea of breathing that usually occurs during sleep. CIH has been linked to various health problems, including cardiovascular disease (58), cognitive impairment (59), and oxidative stress (60). CIH can cause neuronal damage in the CNS, which induces a response from microglia cells in the brain. For example, CIH can activate microglia, increasing the production of pro-inflammatory cytokines and other factors contributing to neuroinflammation (61). Meanwhile, excessive neuroinflammation caused by microglia activated after CIH can negatively affect brain function (62). After CIH, a significant increase in microglial cell density was observed in the dorsal region of the hippocampus, regardless of age differences (63). The CIH induced the majority of microglia to differentiate into pro-inflammatory phenotypes, leading to upregulated expression of inflammatory cytokines IL-6, IL-1β, and TNF-α. When the pro-inflammatory and anti-inflammatory subtypes of microglia are appropriately regulated, it can alleviate CIH-induced brain injury (64, 65). To summarize, while these findings have revealed alterations in the inflammatory cytokines of microglia in CIH, a more comprehensive analysis of the transcription and protein profiles of polarized microglia in this disease model is still lacking. Therefore, relying solely on indicators of inflammatory cytokines makes it challenging to understand the precise role of microglia in such diseases.

### 2.3 Neonatal hypoxic-ischemic brain injury

The NHIBI is a significant cause of neonatal morbidity and mortality, leading to long-term neurological deficits (66). In recent years, research has focused on the roles of microglia in the pathogenesis of NHIBI (67, 68). Following NHIBI, inflammation-sensitized reactive microglia significantly up-regulate the expression of genes related to pro-inflammatory molecules, such as iNOS, IL-1β, and IL-6 (69). Similarly, Bernis et al. found that microglia, as early vital mediators of the inflammatory response, polarize towards the predominant pro-inflammatory phenotype shortly after NHIBI (70). The NHIBI leads to a profound activation and proliferation of microglia and strongly induces miR-210 upregulation in activated microglia. Intrinsically, miR-210 can promote the activation of microglia towards a pro-inflammatory phenotype, thereby enhancing the expression of related proinflammatory cytokines (71). Microglia have also been found to induce NLRP-3/caspase-1/GSDMD axis-mediated canonical pyroptosis in NHIBI (72). Inhibition of the colony-stimulating factor 1 receptor (CSF1R), which is crucial for microglial survival, can effectively regulate the inflammatory response of microglia, alleviating excessive neuroinflammation and brain injury resulting from acute cerebral hypoxic-ischemia (73). However, microglia in NHIBI do not exhibit only one response trend. For example, microglia transformed an inflammatory, amoeboid phenotype to a restorative, anti-inflammatory phenotype within 24 to 48 hours of treating extracellular vesicles in brain tissue after the NHIBI (74). Notably, the interactions among different subtypes of microglia following NHIBI appear to be a complex, time-dependent continuum involving early pro-inflammatory and later anti-inflammatory subcluster responses (75). The balance between microglial activation and neuroprotection is crucial in determining the outcome of NHIBI.

In summary, stroke, CIH, and NHIBI reveal subpopulation transformation and functions of microglia at different levels of injury and stages (**Table 1**). Interestingly, the inflammatory response of microglia was weaker in older samples of hypoxic-ischemic brain injury. This may be related to the accumulation of chronic inflammation in the body caused by aging. With the increase of age, the body has been exposed to a chronic inflammatory environment for an extended period and has developed a certain tolerance, which can result in a weakened inflammatory response (76). For example, some studies provide evidence that there are age-specific immune differences present in the brain (77, 78). In conclusion, reactive microglial response is dependent on the degree of injury and the stage of the CNS development.

**Table 1:** Microglial expression profiles and functions under various pathological conditions.

Diseases	Diseases Stage	Age Group	Differential Expression Gene	Function of Microglia	References
**Stroke**	Acute Phase (After 24 h-3 day)	8,9,10,12-weeks- male C57BL/6J mice	Id2↑;Gadd45b↑; Ccl4↑; Cd83↑; Rcan1↑	Phagocytic vesicles	(45)
			TNF-α↑; IL-6, 2↑	Inflammatory response	
			Prdx1↑; Srxn1↑; Txn1↑; Mt1, 2↑	Resist oxidative stress	(53)
			SPP1↑; Fth1↑; Cd63↑; Itga5↑	Neuroprotective and repairing	
			Hsp90aa1↓; Hspa1a↓; Hspa1b↓	Heat shock protein	
			iNOS↑; Cd11b, 16, 32, 86↑	Inflammatory response	(52)
	Acute Phase (After 3 day)	3-month-male spontaneously hypertensive rats	Mki13↑; Gpnmb↑; Lgals3↑	Microglia proliferation and activation	(55)
			Cxcr4↑ COX-2↑	Inflammatory response	
			Fabp4,5↑	PPAR signaling	
			Ifna1↑; Bst2↑	Interferon response	
		12-month-male spontaneously hypertensive rats	Gpnmb↑; Lgals3↑	Microglia activation	
			Cxcr4↓; COX-2↓	Inflammatory response	
			Fabp4,5↑	PPAR signaling	
			Ifna1↓; Bst2↓; Irf7↑	Interferon response	
	Sub-acute Phase (After 5 day)	10-weeks-male C57BL/6J mice	Cspg4↑; Cst7↑; Chst2↑; Cxcl10↑	Inflammatory response	(56)
			Ccl2, 3, 4, 6, 8, 12, 10, 16↑	Chemokine	
			Spp1↑; Cspg4↑; Gpnmb↑; Col1a1, a2↑; Col5a1, a2↑; Col4a2↑	Neural repairing	
			Clec7a↑; Ptger4↑; IL-1b, 1m, 2rg↑; Itgax↑; C5ar1↑	Cell–cell interactions	
		18-months- male C57BL/6J mice	Cspg4↑; Cst7↑; Chst2↑; Cxcl10↑	Inflammatory response	
			Ccl6↓	Chemokine	
			Spp1↑; Cspg4↑; Gpnmb↑; Col1a1, a2↑; Col5a1, a2↑; Col4a2↑	Neural repairing	
	Chronic Phase (After 14 day)	3-month-male spontaneously hypertensive rats	Fabp4,5↑	PPAR signaling	(55)
			Irf7↑	Interferon response	
			C3,4a↑	Complement cascade	
		12-month-male spontaneously hypertensive rats	Fabp4,5↑	PPAR signaling	
			Irf7↑	Interferon response	
			C3,4a↑	Complement cascade	
		10-weeks- male C57BL/6J mice	Ttr↓; Enpp2↓; Hspa1b↓	lipid signaling	(57)
			Adam8↑	Cell–cell interactions	
			Mrc1↓	Inflammatory response	
			Spp1↑Vegf↑; IGF-1↑	Neural repairing and angiogenesis	
			Ccr2↑; Plaur↑; Tlr2↑; Axl↑; Itgax↑	Cell recruiting	
		18-months-male C57BL/6J mice	Ttr↑; Enpp2↑; Hspa1b↑	lipid signaling	
			Adam8↓	Cell–cell interactions	
			Mrc1↓	Inflammatory response	
			Spp1↑	Neural repairing	
**Chronic Intermittent Hypoxia**	Lasted for 12 weeks for CIH	BALB/c mice	IL-1β, 6↑; TNF-α↑; iNOS↑	Inflammatory response	(64)
		Sprague-Dawley rats	IL-1β, 6↑; TLR4↑; Cox-2↑; TNF-α↑; iNOS↑	Inflammatory response	(65)
**Neonatal Hypoxic- Ischemic injury**	After 6h; 24 h	9-day-both sexes C57BL/6 mice	IL-1β, 6, 10↑; CD86↑; Fizz1↑; Arg1↑	Inflammatory response	(75)
			Gal3↑	Cell–cell interactions	
	After 24 h	7-day-both sexes Wistar rat pups	iNOS↑; IL-1β, 6↑; TGF-β↑; NLRP3↑	Inflammatory response	(69)

↑ indicates up-regulated gene, ↓ indicates down-regulated gene.

## 3 Signaling pathways regulating microglial response in hypoxic-ischemic injury

Microglia mediated neuroinflammation is a complex process. In particular, multiple signaling pathways and transcription factors regulate microglia responses in hypoxic-ischemic injury, including MAPK/NF-κB, JAK2/STAT3, mTOR, CSF1/CSF1R, and PPAR-γ related pathways.

### 3.1 MAPK/NF-κB

The activation of nuclear factor kappa-B (NF-κB) in microglia plays a major role in the pathogenesis of hypoxic-ischemic brain injury. NF-κB, as an inflammatory factor, seems to be involved in the immune response of microglia cells under inflammatory conditions. The overactivation of NF-κB is believed to be a major cause of brain injury, and prophylactic inhibition of NF-κB provides significant neuroprotection against inflammation (79, 80). In microglia, activation of NF-κB transcription is regulated by the SUMOylation and de-SUMOylation of NEMO mediated by SENP1 (81). The mitogen-activated protein kinase (MAPK) pathway is also closely related to the NF-κB pathway. When microglia cells are in oxygen-glucose deprivation, reactive oxygen species (ROS) are produced to activate p38-MAPK/NF-κB pathway signaling (82). Notably, the phosphorylation of MAPK can lead to downstream phosphorylation of NF-κB and transcription of related genes, which is a process that has been associated with microglia-mediated inflammatory responses (83). Additionally, the NF-κB subunit p65 also contributes to the level of Hif-1α mRNA and protein expression (84). When the NF-κB/HIF-1α signaling pathway is inhibited, hypoxia-induced microglia injury is alleviated (85, 86). Endogenously produced hydrogen sulfide (H2S) can also have a similar neuronal protective effect via inhibiting iNOS, NF-κB, ERK, and p38 MAPK signaling pathways (87). Pro-inflammatory factors such as TNF-α, IL-1, and IL-6 are regulated by NF-κB p65 signaling (88). Interestingly, TLR4 expression in hypoxic microglia also depends on the production of inflammatory mediators mediated by HIF-1α/NF-κB related pathways (89). Analgecine was found to inhibit ischemia-induced pro-inflammatory microglial response and promote anti-inflammatory effects via TLR4/MyD88/NF-κB inhibition (90). In summary, up-regulated NF-κB as a pro-inflammatory molecule is important for microglia proinflammatory activation during in hypoxic ischemic brain injury.

### 3.2 JAK2/STAT3

Signaling transducers and transcriptional activators 3 (STAT3) are members of the STAT protein family that mediate stress-related signaling in cells. The Janus kinase 2 (JAK2) is a member of the intracellular non-receptor tyrosine kinases family, and it mediates signaling for cytokine production and is transmitted through the JAK/STAT signaling pathway. The JAK/STAT pathway is activated when cytokines such as IL-6 bind to their corresponding cell surface receptors, triggering the activation of Janus kinases (JAKs) (91). The JAKs then phosphorylate the receptors, which recruit downstream signaling molecules such as STATs (92). The activated STATs translocate to the nucleus and regulate gene expression, leading to various cellular responses such as cell growth, differentiation, and immune responses. In particular, in hypoxic-ischemic brain injury, the JAK2/STAT3 pathway plays a critical role in microglia activation and neuroinflammation (93, 94). This pathway may be a therapeutic target for excessive neuroinflammation after hypoxic-ischemic brain injury. For example, STAT3 activates microglia to increase TNF-α expression, which causes reactive oxygen species (ROS) levels in neuronal cells to increase neuronal apoptosis (95). In hypoxic-ischemic stroke, homocysteine further enhances the activation of STAT3 and the production of inflammatory cytokines such as TNF-α and IL-6 in microglia (96). Conversely, atractylenolide III reduces the production of inflammatory cytokines such as IL-6, IL-β, and TNF-α by inhibiting JAK2/STAT3/Drp1 pathway mediated mitochondrial fission in hypoxic-ischemic microglia injury (97). Meanwhile, inhibiting JAK2 and reducing STAT3 phosphorylation. Ruxolitinib was found to suppress the expression of NLRP3 inflammation-related proteins and multiple pro-inflammatory cytokines in the ischemic cortical penumbral zone, as reported by (98). Interestingly, recent studies have shown that the JAK2/STAT3 pathway induces microglia to transform into pro-inflammatory tendencies, releasing pro-inflammatory factors (99). Together, most evidence supports a pro-inflammatory role for STAT3 signaling in reactive microglia.

### 3.3 mTOR

Mammalian target of rapamycin (mTOR) is a serine/threonine protein kinase that acts as a key regulator of cell metabolism, growth, and survival (100). mTOR has been shown to play important roles in microglia response to hypoxic-ischemic injury. The activation process of microglia is accompanied by the phosphorylation of mTOR (101, 102), leading to neuroinflammation (103). On the contrary, when mTOR is inhibited, excessive neuroinflammation and neuronal death can be effectively prevented (104). In addition, mTOR may suppress microglial autophagy by inhibiting ULK1, therefore attenuating neuroinflammation and its associated pathologies (105). mTOR appears to be closely associated with the phenotypic transformation of microglia to pro-inflammatory tendencies (106). Interestingly, in the hypoxic-ischemic stroke rat model, the knockdown of PLXNA2 facilitates the transformation of microglia from a proinflammatory to an anti-inflammatory phenotype, which is mediated by the mTOR/STAT3 signaling pathway (107). PI3K/Akt acting upstream of mTOR is also closely related to microglia activation (108), and its suppression inhibits neuroinflammation (109). Under *in vitro* hypoxia conditions, the PI3K/Akt/mTOR pathway is activated by the hypoxic-inducible factor 1α(HIF-1α), resulting in upregulation of iNOS expression in microglia (110). Overall, mTOR is a critical regulator of microglial inflammatory balance, and its dysregulation is implicated in various neurological diseases. The essence of the issue is how to maintain a balance in mTOR regulation during hypoxic-ischemic injury.

### 3.4 CSF1/CSF1R

Colony-stimulating factor 1 (CSF1) is a key cytokine that promotes the survival, proliferation, and differentiation of microglia (111, 112). It is produced by neurons (112) and astrocytes (113). It binds to its receptor CSF1R (colony-stimulating factor 1 receptor) on the surface of microglia to activate downstream signaling pathways that regulate microglia function (114–116). Microglia survival, proliferation, and response rely heavily on the critical role of CSF1R. A recent study showed that CSF1R appears to be involved in redox status-related signaling of microglia (117), and inhibition of CSF1R by ki20227 treatment in the microglia may have a negative clinical effect following hypoxic-ischemic stroke (118). A similar CSF1R antagonist, Pexidartinib (PLX3397), also reduced the proliferation of microglia, but brain injury from hypoxia seems to be alleviated after PLX3397-treatment (73). The variability in results may be attributed to the dynamic response of microglia during inflammation. Therefore, it would be unwise to assume that microglia are solely detrimental to the niche. Meanwhile, CSF1R activation with recombinant human CSF1 ameliorated neuroinflammation after HIBI via CSF1R/PLCG2/PKCϵ/CREB signaling pathway in microglia (119). In addition, the CSF1/AMPK pathway triggers microglia activation leading to autophagy and promoting microglia-derived factors secretion (120). As a cytokine targeting microglia, CSF-1 needs more basic and clinical research to better understand how it regulates the balance of neuroinflammation and neuroprotection.

### 3.5 PPAR-γ

Peroxisome proliferator-activated receptor gamma (PPAR-γ) is a nuclear receptor that regulates neuroinflammatory and neuroprotective in microglia (121, 122). PPAR-γ is activated during the anti-inflammatory response of microglia (123). The PPAR-γ related pathways are activated to regulate the dynamic changes of pro-inflammatory and anti-inflammatory factors and induce the response of microglia toward anti-inflammatory trends (124). Upregulation of PPAR-γ also appears to inhibit signaling in the NFKB pathway and regulate the expression of transcription factors, such as Nrf2 and CREB, and their downstream pro-inflammatory/anti-inflammatory genes (125). Interestingly, PPAR-γ is also involved in the mechanism of neuroprotective effects in hypoxic-ischemic brain injury (126). Especially, the PPARγ/Nrf2/CREB pathways in microglia have been demonstrated to inhibit oxidative stress, inflammation, and apoptosis, thereby substantially mitigating hypoxic-ischemic brain injury (127). Similarly, the response trend of microglia toward anti-inflammatory subtypes induced by oxygen-glucose deprivation was also regulated via PPARγ related pathway (128). These findings suggest that PPAR-γ is important in regulating microglia activation and function, especially the anti-inflammatory function.

**Figure 1:**
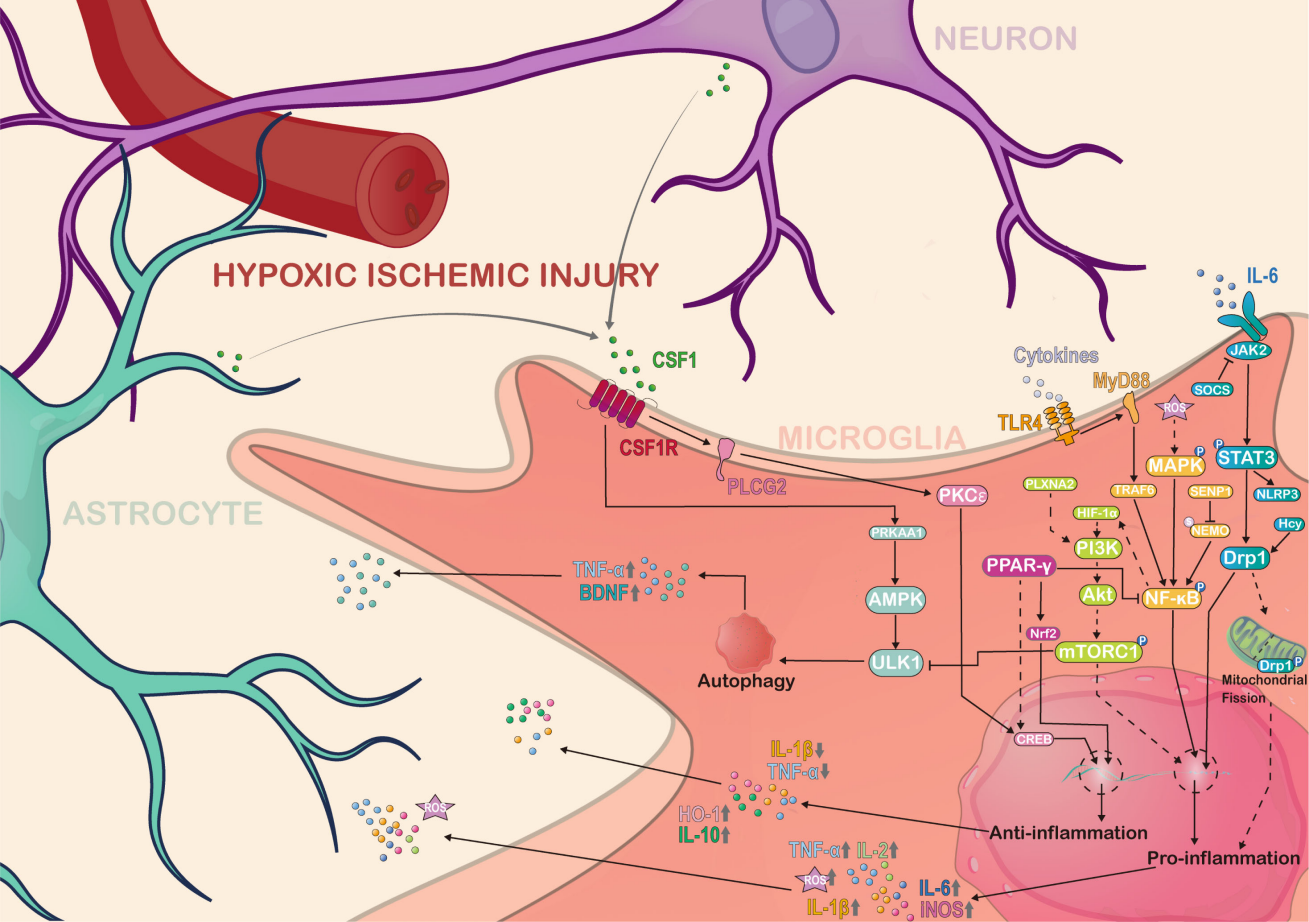
Molecular complexity and regulatory pathways of microglia in hypoxia ischemic brain injury. Microglia are complex regulated by various pathways in hypoxic-ischemic brain injury, and also interact with other cells and molecules in the central nervous system to secrete various cytokines, chemokines and neuroregulatory molecules to regulate the homeostasis of the CNS niche. Solid-lined arrows represent the mechanisms previously examined in the literature. Dotted arrows indicate potential mediating pathways that have not been fully investigated from previous work. AMPK, Adenosine 5’-monophosphate (AMP)-activated protein kinase; Akt, known as protein kinase B; PRKAA1, Protein Kinase AMP-Activated Catalytic Subunit Alpha 1; PKCϵ, Protein kinases C ϵ isoforms; ULK1, Unc-51 Like Autophagy Activating Kinase 1; MAPK, Mitogen-Activated Protein Kinase; CSF1, Colony Stimulating Factor 1; CSF1R, Colony Stimulating Factor 1 Receptor; PLCG2, Phospholipase C Gamma 2; NF-κB, Nuclear factor kappa-B; S, Small ubiquitin-like modifier; SENP1, SUMO Specific Peptidase 1; NEMO, NF-kappa-B essential modulator; PPAR-γ, Peroxisome proliferator-activated receptor gamma; CREB, Cyclic AMP response element binding protein; Nrf2, Nuclear factor erythroid 2-related factor 2; Hif-1α, Hypoxia-inducible factor 1 alpha; TRAF6, Tumor necrosis factor receptor-associated factor 6; MyD88, Myeloid differentiation primary response 88; ROS, Reactive oxygen species; TLR4, Toll-like receptor 4; PI3K, Phosphatidylinositol 3-kinase; SOCS, Suppressor of cytokine signaling; JAK2, Janus kinase 2; NLRP3, NLR family pyrin domain containing 3; STAT3, Signal transducer and activator of transcription 3; Drp1, Dynamin-related protein 1; TNF-α, Tumor necrosis factor alpha; BDNF, Brain-derived neurotrophic factor; IL, Interleukin; iNOS, Inducible nitric oxide synthase; HO-1, Heme oxygenase 1. This figure was created by Adobe Illustrator software.

In summary, the multifaceted functions of microglia in hypoxic-ischemic injury are regulated by various signaling pathways and transcription factors. Among these pathways, the pro-inflammatory response of microglia is associated with the regulation mechanism of MAPK/NF-κB, JAK2/STAT3, and mTOR, while the anti-inflammatory response of microglia is linked to the PPAR-γ pathway. In addition, the CSF1/CSF1R pathway, which plays a vital role in microglia survival, has also been implicated in the regulation of microglia activation (**Figure 1**). Therefore, the changes of different signaling pathways determines the molecular complexity of microglia and their functions during hypoxic-ischemic injury.

## 4 Dynamic processes of microglial-mediated remodeling and regeneration in hypoxic-ischemic brain injury

**Figure 2:**
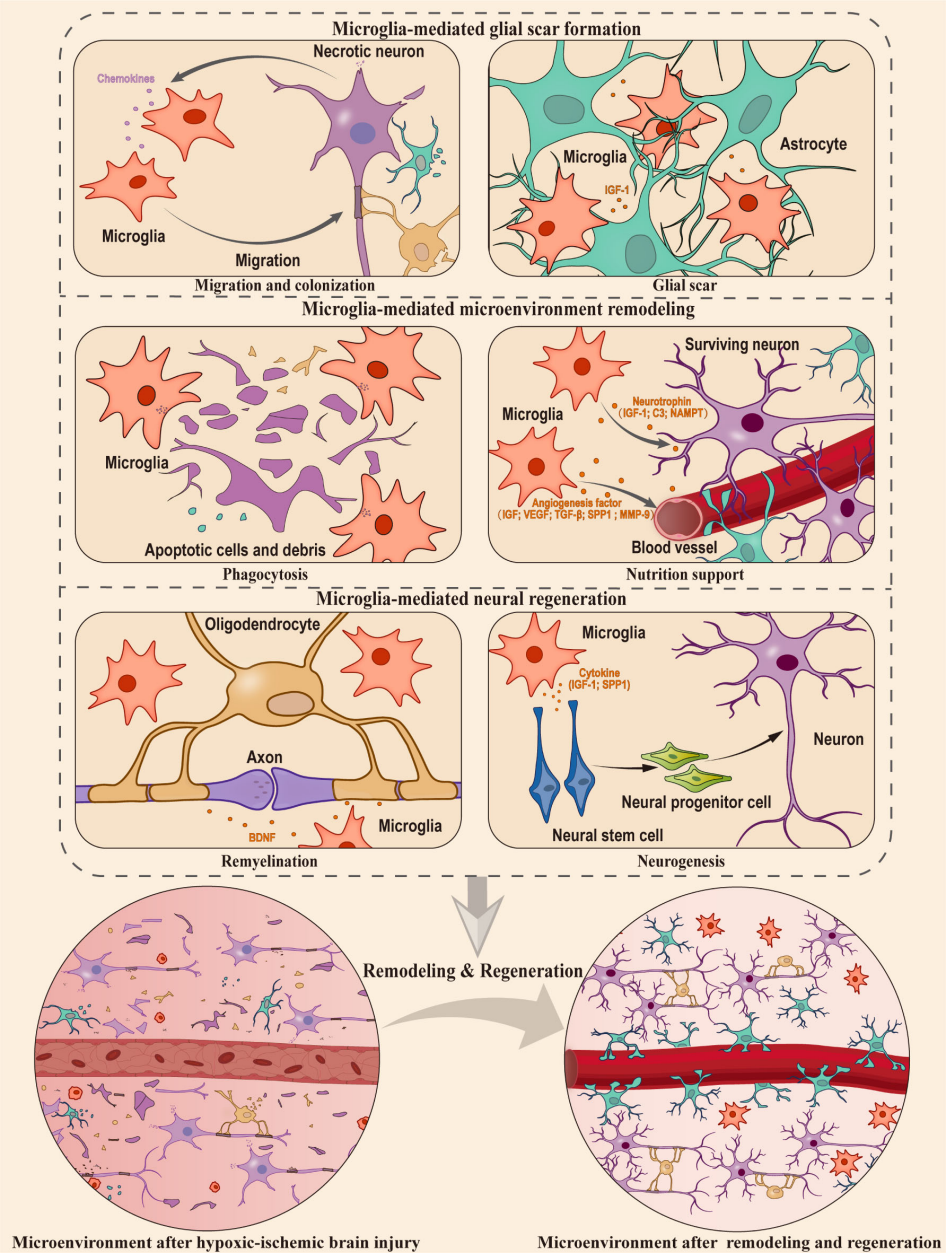
Microglia-mediated microenvironment remodeling and neural regeneration in hypoxic ischemic brain injury. In hypoxic-ischemic brain injury, the CNS sustains significant damage, resulting in the apoptosis and necrosis of numerous neurons and glial cells. Microglia, which act as protectors of the niche, play a crucial role in glia scar formation, microenvironment remodeling, and supporting the processes of neurogenesis and remyelination. The orange cytokines represent those secreted by microglia, and the purple cytokines represent those secreted by neurons. This figure was created by Adobe Illustrator software.

Despite exhibiting negative effects under specific circumstances, there is mounting evidence to support the idea that microglia primarily serve a protective function in hypoxic-ischemic brain injury (70, 129, 130). Based on these studies, this section delves deeper into the impact of microglial cell activation following a hypoxic-ischemic injury on neural tissue remodeling and regeneration (**Figure 2**), which involved in the following stages (1): Microglia-mediated glial scar formation (2). Microglia-mediated microenvironment remodeling (3). Microglia-mediated neural regeneration.

### 4.1 Microglia-mediated glial scar formation

When brain tissue is damaged, apoptotic cells release ‘find-me’ signals, attracting microglia to the site of cell death within the tissue (131). Although the factors that trigger ‘find-me’ signals may vary depending on the specific type of cell injury or illness, in the case of hypoxic-ischemic brain injury, it’s been found that chemotactic signals released by necrotic neurons can attract microglia to the site of injury rapidly (132). These activated microglia release various cytokines and growth factors, such as insulin-like growth factor 1 (IGF-1), which play a crucial role in the recruitment and activation of astrocytes (133). After the recruitment, microglia and astrocytes work together to establish a complex network of glial processes and extracellular matrix components, ultimately leading to the formation of a glial scar (134). During the occurrence of injury, glial scar serves as a physical barrier, aiding in the isolation of the injured site (135), and the microglia within the glial scar effectively restrict the spread of damage (136) providing a platform for microenvironment remodeling and regeneration processes. In addition, the depletion of microglia cells will inhibit the proliferation of astrocytes mediated by STAT3 phosphorylation, resulting in the disruption of scar formation barrier and increasing neuroinflammation at the injury site (137). However, the role of glial scar in axon regeneration has been a subject of debate for many years. Asya Rolls et al. proposed that the glial scar has a dual nature: it has a beneficial effect during the acute phase of injury, but can prevent chronic or advanced axonal growth (138). Recent findings have challenged this idea, revealing that axon growth does not occur when astrocytes in glial scars are selectively eliminated (139–141). Taken together, accumulating evidences suggest that the formation of glial scar mediated by microglia could play a beneficial role at the site of injury.

### 4.2 Microglia-mediated microenvironment remodeling

The phagocytic function of microglia plays a critical role in the process of neural tissue remodeling after injury. Microglia undergo both morphological and molecular changes that boost their ability to perform phagocytosis (142). The replenishment of microglia occurs in large quantities through the proliferation of resident microglia, which migrate to and colonize the site of injury in response to chemotactic signals (71, 143, 144), Microglia distinguish between cells that need to be engulfed and cells that need to be rescued through signals of eat-me and do not eat-me (131). These signals are related to the expression of membrane proteins on apoptotic neurons and the corresponding receptors on microglial membranes (145). When microglia cells recognize apoptotic cells releasing the eat-me signal, they engulf them. The cellular debris is then digested through the gastrosome (146). Conversely, when presented with surviving neurons and other cells releasing do not eat-me signals, microglia support their survival by secreting neurotrophic factors such as IGF-1 and C3 (147). For example, microglia secrete nicotinamide phosphoribosyl transferase (NAMPT) during hypoxia and glucose deprivation (148). This secreted NAMPT exhibits neuroprotective functions (149). Moreover, microglia protect astrocytes by releasing specific cytokines and contribute to the restoration of overall brain homeostasis under hypoxic conditions (150). These evidence suggest that microglia play an essential role in maintaining the microenvironment homeostasis. Angiogenesis is also an important part of microenvironment remodeling. During the angiogenesis process, CellChat analysis of single-cell data indicates that microglia may regulate vascular endothelial cells through the SPP1 and IGF signaling pathways, promoting endogenous angiogenesis (151). Following hypoxic-ischemic injury, activated microglia by LPS stimulation also facilitate vascular endothelial growth factor (VEGF) secretion and the migration of retinal microvascular endothelial cells (152). In addition, microglia can transit to a neuroprotective state, activating the TGF-β1-dependent Smad2/3 pathway through the secretion of extracellular vesicles. This process inhibits the apoptosis of hypoxic neurons and promotes angiogenesis post-injury (153). Similarly, OGD-pretreated microglia have been observed to secrete VEGF, TGF-β, and matrix metalloproteinase-9 (MMP-9). When transplanted into the ischemic core boundary, these microglia foster angiogenesis and axon growth (154). Furthermore, a subset of microglia conducive to vascular regeneration after hypoxic-ischemic brain injury can be induced through treatment with certain drugs (155, 156). Conversely, the depletion of microglia heightens vascular leakage in the spinal cord during chronic mild hypoxia (157), underscoring the critical role of microglia in maintaining vascular integrity. In summary, microglia play multiples roles in the remodeling of the microenvironment by surveillance, phagocytosis, and facilitating angiogenesis.

### 4.3 Microglia-mediated neural tissue regeneration

After the microenvironment remodeling is completed, the neural tissue enters the phase of regeneration. This stage encompasses two crucial events: neurogenesis and remyelination. To provide a comprehensive understanding of the impact of microglia cells on neural tissue regeneration, we will summarize the roles of microglia during the processes of neurogenesis and remyelination in the following paragraphs.

#### 4.3.1 Neurogenesis

Neurogenesis refers to the process of neuronal generation, involving the differentiation of neural progenitor cell (NPC), migration and maturation of neurons, axonal growth, and synaptic formation, ultimately establishing a fully functional neural network (158, 159). Particularly, there is compensatory neurogenesis after hypoxic-ischemic brain injury (160, 161). This process requires the involvements of appropriate cytokines, neurotrophins, and supporting niche cells (162, 163). Therefore, the unique composition of the neurogenic niche plays a pivotal role in regulating neurogenesis. Hypoxic-ischemic brain injury triggers molecular changes in the niche cells, activating niche signals that influence neurogenesis (164). Microglia, as critical neural niche cells, play a crucial role in regulating neurogenesis. For example, when microglia undergo phagocytosis state, their transcriptomes and secretomes show a tendency to promote neurogenesis (165). They have been proven to effectively support neural stem cell (NSC) proliferation through the secretion of various factors following hypoxic-ischemic injury, such as IGF-1 (166, 167) and SPP1 (168). The hypoxic-ischemic injury activated microglia can also impact NPC proliferation through direct cell-cell interactions (169). A recent study used single-cell sequencing to construct myeloid cell composition map of the periinfarction area after ischemic stroke in rats, and further distinguished microglia state conducive to neural regeneration, the underlying mechanism of which can be attributed to SOX2 and its involvement in the regulation of Akt and CREB signaling pathways (170).Studies have shown that the knockout of the HCAR1 gene inhibits the activation of microglia, thereby weakening neurogenesis after hypoxic-ischemic brain injury (171). *In vitro* experiments showed that NSC cultured in a conditional medium with anti-inflammatory microglia subtypes exhibited better cell survival, stronger migration ability, and lower astrocyte differentiation ability (172). Interestingly, the impact of microglia on the proliferation of NSC is dependent on their mutual interactions with each other since the conditional medium directly collected from primary microglia appears to have no effect on NSC proliferation (166, 173). Conversely, experiments using transwell contact culture systems or conditional medium of microglia cells that directly interact with NSCs both showed a positive effect on neurogenesis (166, 169, 174, 175). Meanwhile, neural stem cells also induce microglial response through CXCL/CXCr-related chemokine signaling (176, 177). Moreover, repopulating microglia in traumatic brain can promote adult neurogenesis via the IL-6 trans-signaling pathway, and directly improving the survival rate of newborn neurons and supporting cognitive function (178). A recent study has also shown that IL-4 driven microglia in the hippocampus transform to Arg1^+^ phenotype under stress, and the microglia in this state promote neurogenesis through the BDNF signaling pathway (179). Therefore, microglia do not behave phenotypes that are beneficial to neurogenesis without receiving the signaling from NSC and other cells in the niche. It is worth to explore and clarify the communicating factors involved in the crosstalk between microglia and neurogenic niche cells.

#### 4.3.2 Remyelination

Myelin sheaths, generated by oligodendrocytes, play a crucial role in the CNS. They are responsible for maintaining the structural integrity of neurons, providing neurotrophic factors, and facilitating electrical signal transmission, thereby promoting overall neural health and cognitive function (180–182). Remyelination refers to the process in which newly differentiated oligodendrocytes form myelin sheaths around demyelinated axons, reconstructing efficient electrical impulse conduction, neural health, and motor function (183). Although the impact of microglia on myelin sheath formation during normal development is minimal, they play a crucial role in maintaining myelin homeostasis. They prevent excessive myelin phospholipid growth and demyelination while maintaining the existing balance of myelin lipids (184). Interestingly, microglia can read the physiological state of neurons through contact with ranvier, thus changing their own state to regulate neuronal survival and remyelination (185). In addition, dong et al. found that microglia can reduce the damage caused by oxidative stress after demyelination by clearing oxidized phosphatidylcholines (186). During the phase of neural regeneration, activated microglia recruit oligodendrocyte precursor cell (OPC) and promote their differentiation, thus supporting the completion of remyelination (187). Particularly after hypoxic-ischemic brain injury, the activation of microglia significantly increases during the process of myelin sheath formation, influencing the differentiation of oligodendrocytes (188). Depletion of microglia leads to a significant downregulation of myelin formation markers, such as Olig2, Myrf, and Nkx2.3, exacerbating demyelination (189), and affecting the differentiation of oligodendrocytes (190). Interestingly, microglia express genes associated with cell growth-supporting, such as IGF-1, SPP1, Csf1, and genes related to lipid metabolism, such as Abca1, Abcg1, Apoe, Apoc1, and Lpl. The high expression of these cell growth-supporting genes in microglia may have effects on other cells. Further research is needed to address this question. This indicates their important role in the repair process (191). Notably, SPP1 has been implicated in the endogenous repair process following ischemic-hypoxic brain injury in knockout mice (192). Further research has revealed that osteopontin (SPP1) produced by Treg cells promotes microglia-mediated remyelination by interacting with the integrin β1 (ITGβ1) receptor on the surface of microglia (129). However, the exact proteins secreted by microglia and the OPC receptors involved in remyelination need to be identified. Overall, the role of microglia in the process of remyelination after ischemic hypoxic brain injury is crucial, and further understanding of the regulatory mechanisms involved is necessary.

## 5 Conclusions

As native immune cells in the CNS niche, microglia can perceive changes in the microenvironment and respond accordingly. Microglial response is a complex process during hypoxic-ischemic brain injury. The activated microglia exhibit diverse phenotypes and their heterogeneity is influenced by different pathological progressions. A better understanding of the dynamic response of microglia in hypoxic-ischemic brain injury is necessary to dissect their specific role in shaping the microenvironment of the CNS after brain damage.

This review comprehensively summarizes the dynamic changes of microglia in chronic intermittent hypoxia, neonatal hypoxic-ischemic brain injury, and ischemic stroke. It also analyzes the dynamic transcriptional profiles during different pathological stages. We provide an overview of the signaling pathways and cytokine release mechanisms associated with microglia in neuroinflammation. Furthermore, we detail the process of microglia-mediated microenvironment remodeling and neural regeneration.

Based on the role of microglia in mediating microenvironment remodeling and neural regeneration, it is necessary to further explore how microglia influence other cells in the microenvironment through cell-to-cell interactions. Moreover, when microglia release factors that influence other cells, it is crucial to identify the target cells, their receptors, as well as the potential mechanisms and factors involved. These studies will enable us to gain a more comprehensive understanding of the interactions among microenvironmental cells in the CNS and the processes of neural repair after injury.

## Author contributions

HQ: Visualization, Writing – original draft. RZ: Funding acquisition, Supervision, Writing – review & editing.
